# The Danube Fish Database: documenting species distributions across a major European river basin

**DOI:** 10.1038/s41597-026-07249-5

**Published:** 2026-05-27

**Authors:** Yusdiel Torres-Cambas, András Ambrus, Miklós Bán, Bálint Bánó, Anthony Basooma, Vanessa Bremerich, Florian Borgwardt, Maša Čarf, Irina Cernisencu, Gorčin Cvijanović, István Czeglédi, Sami Domisch, Tibor Erős, Zoltán Fehér, Vivien Füstös, Juergen Geist, Thomas Hein, Milica Jaćimović, Sonja C. Jähnig, Béla Kiss, Maroš Kubala, Klaudija Lebar, Borislava Kostadinova Margaritova, Matej Marušić, Paul Meulenbroek, Stoyan Dobrev Mihov, Attila Mozsár, Zoltán Müller, Christoffer Nagel, Iulian Nichersu, Dušan Nikolić, Sandi Orlić, Joachim Pander, Polona Pengal, Marina Piria, László Polyák, Bálint Preiszner, Simon Rusjan, Márton Sallai, Zoltán Sallai, Péter Sály, Andrea Samu, Brigitte Sasano, Astrid Schmidt-Kloiber, András Sevcsik, Marija Smederevac-Lalić, András Specziár, Twan Stoffers, Zoltán Szalóky, Renáta Szita, Gábor Takács, Péter Takács, Maxim Teichert, Milcho Todorov, Balázs Tóth, Teodora Trichkova, Damir Valić, Zoltán Vitál, Martin Tschikof

**Affiliations:** 1 Leibniz Institute of Freshwater Ecology and Inland Fisheries, Department of Community and Ecosystem Ecology, Müggelseedamm 310, D-12489 Berlin, Germany; 2 Universidad de Oriente, Department of Biology, Patricio Lumumba sn, Santiago de Cuba, Cuba; 3 Independent Researcher, Jurisich u. 16., H-9495 Kópháza, Hungary; 4 HUN-REN DE Behavioural Ecology Research Group, University of Debrecen, Egyetem tér 1, H-4010 Debrecen, Hungary; 5 HUN-REN Balaton Limnological Research Institute, Tihany, H-8237 Hungary; 6 BOKU University, Institute of Hydrobiology and Aquatic Ecosystem Management, Gregor-Mendel-Strasse 33, 1180 Vienna, Austria; 7 Christian Doppler Laboratory for Meta Ecosystem Dynamics in Riverine Landscapes, BOKU University, Vienna, Institute of Hydrobiology and Aquatic Ecosystem Management, 1180 Vienna, Austria; 8 Fisheries Research Institute of Slovenia, Spodnje Gameljne 61a, 1211 Ljubljana - Šmartno, Slovenia; 9 Danube Delta National Institute for Research and Development, 165 Babadag street, Tulcea, 820112 Romania; 10 University of Belgrade - Institute for Multidisciplinary Research, National Institute of the Republic of Serbia, Kneza Višeslava 1, 11030 Belgrade, Serbia; 11 WWF Hungary, Álmos vezér útja 69/A, 1141 Budapest, Hungary; 12 Department of Hydraulic and Water Resources Engineering, Faculty of Civil Engineering, Budapest University of Technology and Economics, H-1111 Budapest, Hungary; 13 HUN-REN–BME Water Management Research Group, Budapest, H-1111 Hungary; 14 Aquatic Systems Biology Unit, Technical University of Munich (Technische Universität München), Mühlenweg 22, 85354 Freising-Weihenstephan, Germany; 15 Geography Department, Humboldt-Universität zu Berlin, 10099 Berlin, Germany; 16 BioAqua Pro Ltd., Debrecen, H-4032 Hungary; 17 Water Research Institute, Nábr. arm. gen. L. Svobodu 5 (7), 812 49 Bratislava, Slovakia; 18 University of Ljubljana, Faculty of Civil and Geodetic Engineering, Jamova 2, 1000 Ljubljana, Slovenia; 19 WWF Bulgaria, 147 Knyaz Boris I Str., Floor 1, Sofia, 1000 Bulgaria; 20 DANUBEPARKS, Danube River Network of Protected Areas, Orth an der Donau, Austria; 21 Ruđer Bošković Institute, Bijenička cesta 54, 10000 Zagreb, Croatia; 22 University of Montenegro, Cetinjski put 2, Podgorica, Montenegro; 23 Institute for Ichthyological and Ecological Research, Business Unit Ljubljana, Staretova ulica 1, 1233 Dob, Slovenia; 24 University of Zagreb Faculty of Agriculture, Zagreb, Croatia; 25 Department of Ecology and Vertebrate Zoology, Faculty of Biology and Environmental Protection, University of Łódź, Łódź, Poland; 26 Research Center for Fisheries and Aquaculture, Hungarian University of Agriculture and Life Sciences, Gödöllo, Hungary; 27 Vaskos Csabak Bt., Békésszentandrás, Hungary; 28 HUN-REN Institute of Aquatic Ecology, Centre for Ecological Research, Budapest, H-1113 Hungary; 29 Federal Agency for Water Management, Institute for Aquatic Ecology and Fisheries Management, Scharfling 18, 5310 Mondsee, Austria; 30 Duna-Ipoly National Park Directorate, Budapest, H-1121 Hungary; 31 Aquaculture biology and Fisheries ecology group, Wageningen University & Research, Wageningen, The Netherlands; 32 Fertő-Hanság National Park Directorate, Sarród, H-9435 Hungary; 33 Institute of Biodiversity and Ecosystem Research, Bulgarian Academy of Sciences, 1 Tsar Osvoboditel Blvd., 1000 Sofia, Bulgaria

## Abstract

The Danube River Basin (DRB) harbors the highest documented fish species richness of any European river, yet native populations face increasing threats from physical infrastructures that impede longitudinal and lateral connectivity, unsustainable fisheries, the introduction of non-native species, and climate change. Spanning across 19 countries, the DRB presents conservation challenges that demand coordinated, transboundary data sharing. The present database compiles and standardizes fish occurrence datasets that have been previously unavailable, fragmented and often restricted by federal agencies, research institutes, and conservation organizations, integrating also data from sources such as the Global Biodiversity Information Facility, the Joint Danube Surveys, the European Fish Index, and national monitoring programmes. It contains 133,131 occurrence records across 114 fish species, representing 30 families and 17 orders, with a temporal range from 1856 to 2024, organized into 39 columns. By supporting fish community conservation, invasive alien species monitoring, and climate impact assessments, this database provides a vital resource for developing evidence-based management strategies in the DRB.

## Background & Summary

The Danube River Basin (DRB) hosts the highest reported fish species richness among European rivers, with more than 100 species, though the exact number varies depending on the source, ranging between 102 and 124 species. It supports around 20% of Europe’s freshwater fish fauna, including nearly 30 endemic species—some restricted to single lagoons or rivers—while unfortunately also harboring a similarly high number of introduced alien species^1–3^. However, extensive industrialization and river regulation since the 19th century have placed significant pressure on native fish communities in the DRB^4^. Anthropogenic impacts, including physical infrastructures that impede longitudinal and lateral connectivity, unsustainable fisheries, the introduction of non-native species, and climate change have collectively contributed to the decline of native fish populations^5–10^. This trend reflects both regional and global patterns, as freshwater fish are the second most threatened animal group in Europe, surpassed only by molluscs, and are among the most endangered vertebrates worldwide^11,12^. Within the DRB, this vulnerability is evident, with approximately 25 native fish species classified as globally threatened^13^.

Beyond ecological concerns, the DRB presents unique governance challenges. As the most international river basin in the world, spanning across 19 countries, it requires coordinated transnational efforts to manage freshwater biodiversity effectively^14^. Differences in administrative frameworks, monitoring standards, and data accessibility create obstacles for conservation planning and large-scale ecological assessments^10^. Consequently, there is a pressing need for harmonized data-sharing initiatives to support evidence-based conservation strategies.

To address the challenges of varying and scattered data sources in this heterogeneous river basin, this database^15^ is an effort to provide fish occurrence data in the DRB open access, complementing repositories, such as the Global Biodiversity Information Facility (GBIF, https://www.gbif.org/), the Joint Danube Surveys (JDS 1-4, https://www.danubesurvey.org/jds4/), and the European Fish Index^16^ (EFI+). The database^15^ was assembled through collaborations with federal agencies, universities, research institutes, NGOs, national park administrations, private sector stakeholders, and independent researchers. Data sources include research and monitoring projects as well as data collected for EU-mandated status assessments. While some of these datasets were previously available (e.g. for Austria^17,18^ and Hungary^19–22^), they often remained difficult to access due to restricted user permissions or language barriers. By consolidating and standardizing these records, this collection enhances the availability and usability of fish biodiversity data across the DRB, fostering informed decision-making for freshwater ecosystem conservation.

The database^15^ provides essential data to address conservation challenges for fish in Europe and the DRB. It can be used to identify suitable habitats for native species in projects aiming at prioritizing habitat reconnection, taking into account estimated dam removal costs and fish pass construction costs, while also minimizing the spread of invasive species^23^. Additionally, it supports monitoring species responses to climate change, aiding in the prediction of vulnerability and the development of adaptive management strategies^24^. The database^15^ is also valuable for tracking non-native species, enabling early detection of range expansions and providing crucial data for risk assessments and control measures to mitigate their spread^25–28^. Furthermore, fish occurrence data can be integrated with spatially explicit threat data, such as land use intensity, pollution, connectivity barriers, invasive species and climate change^29–32^ to prioritize management actions and contribute to the implementation of the European Union Nature Restoration Law aimed at restoring free-flowing rivers^10^. This approach will help determine which actions to implement and where to implement them to effectively achieve biodiversity recovery targets^33^.

## Methods

We compiled the occurrence data for fish species within the DRB from a variety of sources. Contributions included data from Austria^17,18^, Bulgaria, Croatia, Germany^34–38^, Hungary (^19^, BioAqua Pro Ltd., Duna-Ipoly National Park Directorate^22^, Duna-Drava National Park Directorate, Fertő-Hanság National Park Directorate^20,21^, WWF Hungary^20–22^), Romania, Serbia, Slovakia, and Slovenia (given the partners in the DANUBE4all project, https://www.danube4allproject.eu/), as well as from GBIF^39^, the Fourth Joint Danube Survey (JDS4), and the EFI+ database. Further information on datasets containing primary, unpublished data, including sampling methods and contact persons, is provided in Table 1.

**Table 1:** Overview of individual datasets present in the database^15^ that contain primary, unpublished data, including the methods used to collect occurrence records and the corresponding contact persons.

Dataset name	Method	Contact persons
**Sallai-Vital**	Electrofishing and fyke nets. Additional records, particularly for *Hypophthalmichthys* spp., were obtained from rod-and-reel anglers through social media and subsequently validated by ZV.	Márton Sallai, Zoltán Vitál.
**WWF_BG**	Sampling was conducted in the Danube River using drifting trammel nets 100–150 m in length and 2 m in height, with a main net mesh size of 18–20 mm, along transects ranging from 800 to 2,500 m, mainly between May and September during both daytime and nighttime hours.	Stoyan Dobrev Mihov.
**VUVH**	Occurrence records were collected by electrofishing in the Morava, Danube, Little Danube, Váh, Hron, and Ipeľ rivers during surveys conducted between 2015 and 2019.	Maros Kubala.
**UL_FGG**	Occurrence records were collected in the Sava River between 2010 and 2023 using multiple sampling methods, including electrofishing, multimesh nets, traps, angling, and observational surveys.	Klaudija Lebar, Maša Čarf, Simon Rusjan.
**UB-IMSI**	Occurrence records were collected between 1965 and 2024 from the Danube, Tisza, Sava, Velika Morava, Tamiš, Timok, Mlava, Porečka, Pek, Karaš, Nera, Drina, Studva, and other associated rivers and channels using a range of approaches, including electrofishing, stagnant and drift net fishing, and fisheries data; in projects We Pass 1 and 2, fish were captured by net fishing and tagged for telemetry.	Dušan Nikolić, Gorčin Cvijanović, Marija Smederevac-Lalić, Milica Jaćimović.
**RBI-DANUBEPARKS**	Occurrence records were collected between 2003 and 2024 from the Sava River and its backwaters, multiple habitats within Kopački Rit Nature Park, and several Croatian rivers and water bodies (Drava, Kupa, Kupčina, Orljava, Sunja, Una, Lomnica, Odra, Lipnica, Mrtva Odra Meander, and Lonja) using electrofishing, combined electrofishing and net sampling, and recreational sampling.	Damir Valić, Sandi Orlic, Matej Marusic, Marina Piria.
**INCDDD**	Occurrence records were collected in 2018 and 2019 from the Danube River, including the Saint George, Chilia, and Tulcea branches, Sulina branch and the Lakes in the Delta, as well as the Somova–Parcheș Complex, using net fishing.	Irina Cernisencu, Iulian Nichersu.

Dataset names correspond to the values in the datasetName column of the database.

**Fig. 1:**
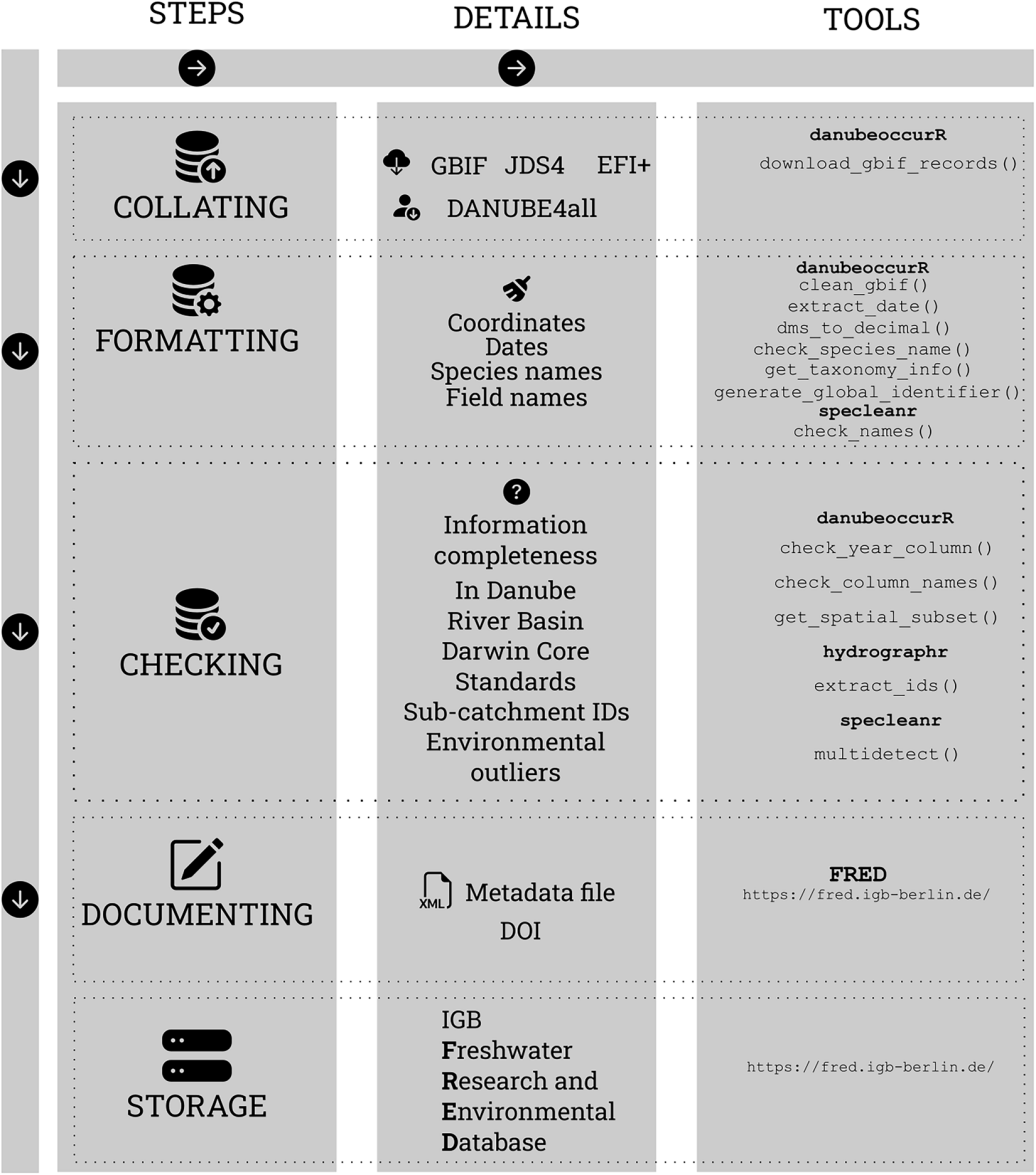
Workflow to compile a database of fish species occurrences from the DRB. The process involves gathering information from various sources, standardizing it to a common format, performing quality checks, and documenting the data using dedicated R packages. The finalized data is stored on an open-source platform for broader accessibility and future use.

In total, 506,290 entries were collected and subsequently subjected to quality checks and cleaning procedures. Following the data collation, formatting, and quality control, we wrapped all the R-code we employed into a new danubeoccurR R-package^40^, to facilitate and streamline the entire process for reproducibility and possible re-use in other regions and datasets. We also incorporated additional R-packages, hydrographr^41^ and specleanr^42^ into the workflow to aid in data manipulation and geospatial analysis (Fig. 1).

For the retrieval of fish species occurrences from GBIF within the DRB, we fetched records based on a preliminary, unpublished checklist compiled by national fish experts from the Fifth Joint Danube Survey (JDS5), which is available as a dataset named species_checklist in the danubeoccurR R-package^40^. This procedure can be applied using the function download_gbif_records() from the danubeoccurR package. We then performed several cleaning and standardization steps to ensure the data’s suitability for modelling. For GBIF data, we removed duplicates and filtered records based on coordinate precision (i.e. number of decimal places in the latitude and longitude values), uncertainty (i.e. radius in meters around the given coordinates within which the actual location is expected to be found), and geographic buffers by applying the clean_gbif() function. This process excluded records with low precision (e.g. less than 3 decimal places) or high uncertainty (above 1000 m) and removed those located near country centroids (within 1000 m), capital city centroids (within 1000 m), and zoo/herbarium centroids (within 1000 m). For other datasets, coordinates were standardized from degrees, minutes, and seconds (DMS) to decimal degrees using the dms_to_decimal() function, while date information was also standardized using the extract_date() function to ensure uniformity across the dataset.

A critical aspect of our data preparation was ensuring taxonomic accuracy across the various data sources. This was achieved by using the check_names() function from specleanr^42^. The function identifies synonyms based on FishBase^43^, corrects spelling errors, and suggests names based on a 90% similarity threshold to ensure the most accurate matches. If automated correction was not feasible, as it was the case for 82 occurrences, we performed manual updates using the check_species_name() function in danubeoccurR^40^. This function provides an interactive interface in RStudio^44^ for users to manually edit species records. Finally, the resulting FishBase species list was compared against the species list in the FreshVerts v1.0 database^45^, which is based on the Eschmeyer Catalog of Fishes (CoF)^46^. FishBase species that were not present in FreshVerts v1.0 (seven species) were double-checked and updated directly against the Eschmeyer Catalog of Fishes using the most up-to-date version of the CoF^46^.

In addition to taxonomic verification, we applied data enrichment steps to complete taxonomic information for each occurrence record. Taxonomic information such as genus, family, and order was populated using the get_taxonomy_info() function. Persistent identifiers (UUIDs) were also assigned to each record using the generate_global_identifier() function to align with Darwin Core and other standards. Additionally, to enable further spatial analysis, sub-catchment IDs were extracted using the extract_ids() function from hydrographr^41^, using the sub-catchment layers from the Hydrography90m global dataset^47^.

Following these procedures, we performed additional data validation steps. We verified column names using the check_column_name() function to ensure conformity with Darwin Core terms. The “year” column was checked for missing values and validated to be numeric and within an acceptable range using the check_year_column() function. Coordinate validation was performed using the check_coordinates() function to confirm that latitude and longitude values were in the correct decimal degree format (WGS84). To ensure that only records within the DRB were retained, we applied the get_spatial_subset() function, which performs a spatial filtering operation to keep only those points falling within a specified polygon. In this case, the polygon represents the DRB and was obtained from the Hydrography90m global dataset^47^. Additionally, potential environmental outliers in the occurrence data were detected and flagged using the multidetect() function from specleanr^42,48^. Identifying environmental outliers is a critical step in preparing data for species distribution models (SDMs), as these outliers can skew model predictions by introducing patterns that do not represent broader ecological trends^48^.

## Data Records

### Repository and Access

The Danube Fish Database^15^ is openly available in the Freshwater Research and Environmental Database (FRED), which is the central data repository of the Leibniz Institute of Freshwater Ecology and Inland Fisheries (IGB), at 10.18728/igb-fred-1078.7 (Fig. 1). The database^15^ is structured to facilitate accessibility and reproducibility, with records provided in standardized formats to ensure compatibility with biodiversity and ecological data frameworks.

### Structure and File Formats

**Fig. 2:**
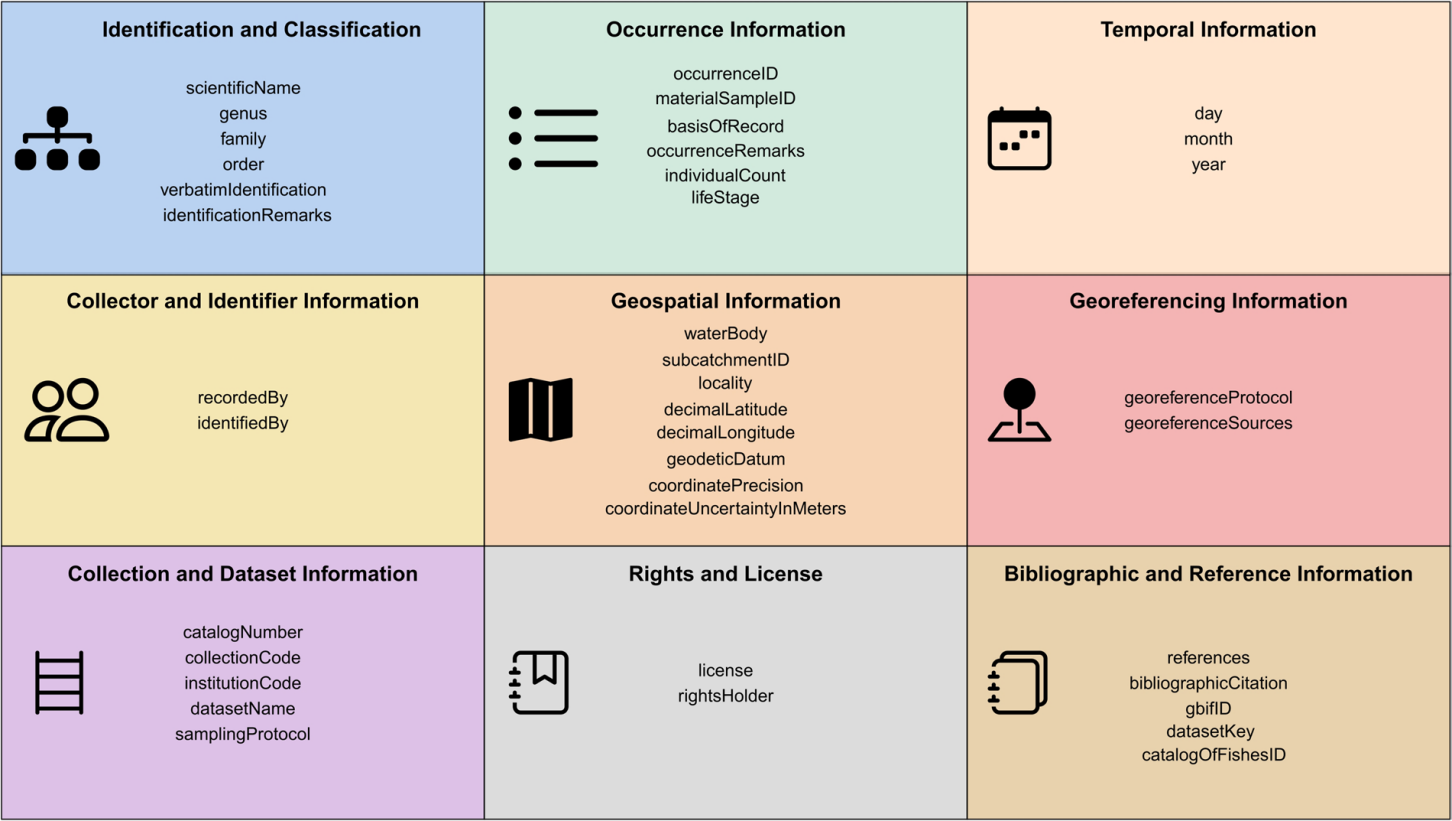
Structure of a database of fish species occurrences from the Danube River Basin. The database fields are organized into nine categories, each represented by a box in the figure. Field names follow Darwin Core Standards.
- metadata.pdf - A metadata file describing column definitions and data sources.

The database^15^ consists of two files, each corresponding to a distinct aspect of fish occurrence records across the DRB. These files are available at 10.18728/igb-fred-1078.7 and are organized as follows:

- occurrence_records.csv - Primary occurrence data. The database of fish species occurrences from the DRB contains 39 fields grouped into nine categories presented in Fig. 2.

## Data Overview

**Fig. 3:**
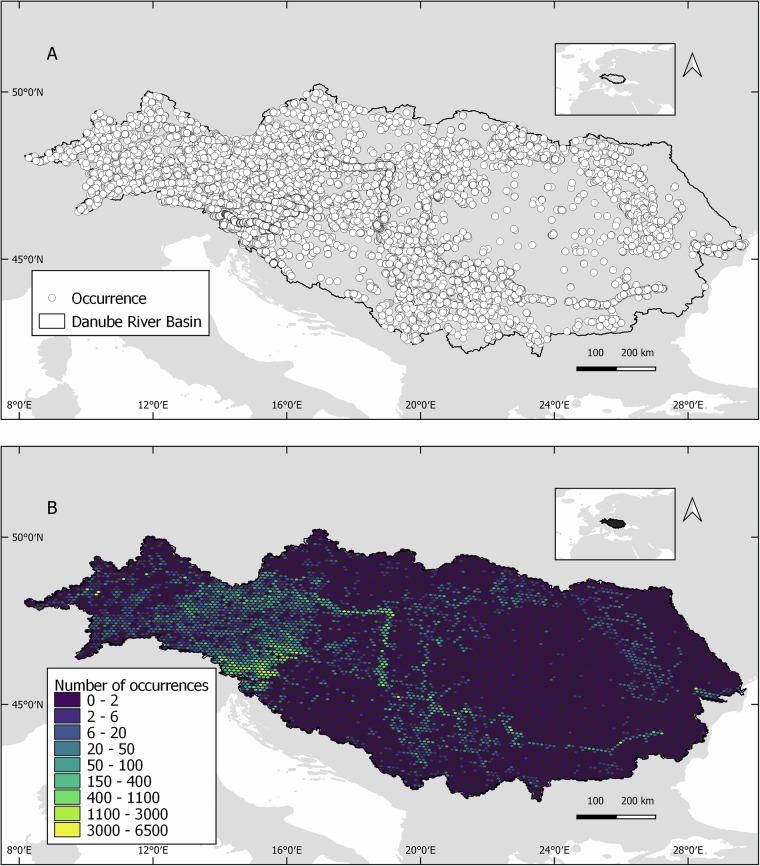
Fish occurrence records across the Danube River Basin are shown for 114 species. Figure 3A illustrates individual occurrence points, while Figure 3B shows the number of occurrences per 10 km^2^ grid cell. The database was compiled from online databases and contributions from DANUBE4all project partners in Austria, Bulgaria, Croatia, Germany, Hungary, Romania, Serbia, Slovakia, and Slovenia, with additional data sourced from GBIF, the Fourth Joint Danube Survey (JDS4), and the EFI+ database. Basin boundaries follow the hydrography90m dataset^47^.

The database^15^ contains 133,131 occurrence records, representing 114 bony fish species across 30 families and 17 orders. Temporal coverage ranges from 1856 to 2024. The median number of records per species is 371, with the most recorded species, the bleak (*Alburnus alburnus*), having 13,582 occurrences. In contrast, the least recorded species *Acipenser naccarii*, *Ictalurus punctatus*, *Ponticola syrman* and *Salmo marmoratus*, each have only one occurrence. Eighty-two percent of the species have ten or more occurrence records. A summary checklist of species and their occurrence counts is provided in Supplementary Table 1. The spatial distribution of records is shown in Fig. 3, while a higher-resolution visualization is available on the IGB-GeoNode (https://geo.igb-berlin.de/layers/geonode:danube4all_fish_occurrence_records), the geospatial information platform of the Leibniz Institute of Freshwater Ecology and Inland Fisheries.

## Technical Validation

To ensure the technical quality of the fish species occurrence database for the DRB, several validation steps were undertaken:

- **Taxonomic Validation:** FishBase^43^ and the CoF^46^ were consulted to ensure alignment with the most up-to-date fish taxonomy, following the semi-automated procedures described in the Methods section. Two species, *Salvelinus umbla* and *Vimba elongata*, are treated as valid taxa in FishBase, but as synonyms of *S. alpinus* and *V. vimba*, respectively, in the CoF. We adopted the CoF treatment as the current valid taxonomic status. For records originally classified as *Salvelinus umbla* and *Vimba elongata*, a note was added to the identificationRemarks and verbatimIdentification fields in the database^15^.
- **Spatial Distribution Validation:** The spatial distribution of species occurrences was assessed to ensure that the recorded locations were geographically plausible and consistent with known habitats for each species. To achieve this, the occurrences were first compared against environmental maps to identify and flag potential environmental outliers (see Methods), and subsequently cross-checked by the respective data provider.
- **Data Completeness and Consistency:** The database was examined for missing values, duplicates, and inconsistencies in key fields such as coordinates, dates, and species names. Gaps in data were identified, and missing or inconsistent records were flagged for review and potential correction.

## Usage Notes

To facilitate data reuse, we provide key recommendations for accessing, processing, and interpreting the database.

- **Data Access and Format:** The database is available in a standard tabular format (CSV) using Darwin Core-compliant terminology to ensure compatibility with biodiversity databases. Users should refer to the metadata file for a detailed description of the column names. For convenience, a custom function named split_and_save_csv() is provided in danubeoccurR^40^ to split the occurrence database into independent datasets.
- **Geospatial Considerations:** Users should be aware that some records may have variable spatial precision, particularly historical occurrences. The function snap_points_on_map() in danubeoccurR^40^ allows users to manually adjust occurrence points for greater precision. Additionally, for applications that require spatial alignment between occurrences and a stream network, we recommend using the snapping functionalities available in the online platform GeoFRESH^49^ for small datasets (e.g. fewer than 100 occurrences), or functions in the R-package hydrographr^41^ for custom workflows and larger datasets.
- **Taxonomic Standardization:** Users are advised to cross-check species names against current taxonomic databases because taxonomic revisions can occur after the database’s publication. To facilitate future taxonomic updates, we also included a column (catalogOfFishesID) containing the Eschmeyer Catalog of Fishes species identification number (spid) for each taxon^46^. Because spid is the identifier used by the Catalog to retrieve species records directly, it provides a stable reference for following taxonomic changes and updating species names in subsequent versions of the database.
- **Data Quality and Potential Limitations:** While efforts were made to standardize and clean the data, users should consider potential sources of bias, including sampling effort variations, taxonomic misidentifications, or incomplete historical records. Some records have been flagged as environmental outliers based on inconsistencies between species occurrence and expected environmental conditions. These flagged records should be reviewed carefully and may require further investigation or validation before inclusion in analyses.
  Spatial bias, resulting from sampling or observation bias, can negatively impact certain applications of occurrence data, such as SDMs^50^. To mitigate sampling bias, spatial thinning can be applied. This technique removes the fewest records necessary to significantly reduce bias while retaining the maximum amount of useful information^51^. Alternatively, in SDMs, it can also be addressed by incorporating covariates that account for accessibility and sampling effort during model calibration^52^.
  Records of *Acipenser naccarii*, should be interpreted with caution. For instance, two specimens of *Acipenser naccarii* recorded in 1995 were sourced from GBIF, originally from the Naturkunde Museum Stuttgart (https://id.smns-bw.org/smns/collection/html/775282/1237352/779394), found in Teichanlage Wöllershof, a pond complex located in Wöllershof, in the Oberpfalz (Upper Palatinate) region of Bavaria, Germany. Since this area is known for its network of fish ponds, it is likely that the record comes from aquaculture.
- **Further species information:** The Danube Fish Database represents a valuable basis for various research fields. Users can flexibly join the dataset to additional information on the biology, autoecology, traits, origin, and conservation status of fish species or communities. A noteworthy example is the freshwaterecology.org database^53^ from where information on autecological characteristics, traits, and geographic origin can be extracted via the fwtraits R-package^54^. Species-level information, including morphological or physiological characteristics can be obtained from FishBase and conservation status from the IUCN Red List via APIs (e.g. (https://api.iucnredlist.org/). Genetic data can be accessed via databases such as BOLD (https://www.boldsystems.org/) or NCBI (https://www.ncbi.nlm.nih.gov/).

## Supplementary information

Supplementary Table 1

## Data Availability

The Danube Fish Database is openly available in the Freshwater Research and Environmental Database (FRED), which is the central data repository of the Leibniz Institute of Freshwater Ecology and Inland Fisheries (IGB), at 10.18728/igb-fred-1078.7.

## Code availability

All functions and example procedures for data sourcing, cleaning, formatting, and quality control of fish species occurrences in the DRB are available through the R package danubeoccurR^40^, which can be installed from GitHub as follows:

# Install remotes package if not already installed

install.packages("remotes")

# Install danubeocurR from GitHub

remotes::install_github("ytorres-cambas/danubeoccurR")

The package includes vignettes that demonstrate key procedures, including:

• **Data Sourcing and Cleaning:** Retrieve occurrence records from GBIF and perform data cleaning. See the vignette General workflow to collate, format, data enrichment and check, sections 2-4 (https://ytorres-cambas.github.io/danubeoccurR/articles/download-and-clean-gbif.html).

• **Taxonomic Standardization:** Verify species names and supplement missing taxonomic information such as genus, family, and order. See the vignette General workflow to collate, format, data enrichment and check, sections 6 and 7 (https://ytorres-cambas.github.io/danubeoccurR/articles/download-and-clean-gbif.html).

• **Adding Additional Information:** Incorporate geospatial data such as administrative names, subcatchment identification numbers and other information such as persistent identifiers for occurrences. See the vignette General workflow to collate, format, data enrichment and check, section 8-10 (https://ytorres-cambas.github.io/danubeoccurR/articles/download-and-clean-gbif.html).

• **Quality Control:** Ensure data completeness and proper formatting, verifying that each record includes at least a species name, date, and coordinates. Also, check column names and coordinate and date format. See the vignette General workflow to collate, format, data enrichment and check (https://ytorres-cambas.github.io/danubeoccurR/articles/download-and-clean-gbif.html).

• **Snapping Points:** Manually adjust occurrence points for improved spatial accuracy. See the vignette Manually snap species occurrence points (https://ytorres-cambas.github.io/danubeoccurR/articles/manually-snap-points.html).

The package is implemented in R (version 4.4.3) and relies on dependencies such as sf^55,56^, tidyverse^57^, rgbif^58^, spcleanr^42^ and CoordinateCleaner^59^, which are automatically installed during package installation. Users can access documentation, report issues, or contribute to development through the GitHub repository https://github.com/ytorres-cambas/danubeoccurR.

Additionally, an example of the procedure used for detecting environmental outliers is available on the specleanr^42^ package’s website. For more details, see the vignette Environmental outlier detection in fish species records (https://anthonybasooma.github.io/specleanr/articles/eOutlier.html).
