# Supplementary material for: The Danube Fish Database: documenting species distributions across a major European river basin
Source: Sci Data. 2026 May 27;13:786. doi: 10.1038/s41597-026-07249-5 (PMC13216254; doi:10.1038/s41597-026-07249-5)
Supplement: Supplementary file 1 — Supplementary Table 1 [file 41597_2026_7249_MOESM1_ESM.pdf]

Supplementary Table 1. List of fish species in a database of species occurrences within the Danube River Basin. The database was compiled from online databases and contributions from DANUBE4all project partners in Austria, Bulgaria, Croatia, Germany, Hungary, Romania, Serbia, Slovakia, and Slovenia, with additional data sourced from GBIF, the Fourth Joint Danube Survey (JDS4), and the EFI+ database.

Species	Family	Order	Occurrences
Abramis brama	Leuciscidae	Cypriniformes	1699
Acipenser baerii	Acipenseridae	Acipenseriformes	5
Acipenser gueldenstaedtii	Acipenseridae	Acipenseriformes	19
Acipenser naccarii	Acipenseridae	Acipenseriformes	1
Acipenser ruthenus	Acipenseridae	Acipenseriformes	312
Acipenser stellatus	Acipenseridae	Acipenseriformes	35
Alburnoides bipunctatus	Leuciscidae	Cypriniformes	4852
Alburnus alburnus	Leuciscidae	Cypriniformes	13582
Alburnus chalcoides	Leuciscidae	Cypriniformes	3
Alburnus mento	Leuciscidae	Cypriniformes	28
Alburnus sarmaticus	Leuciscidae	Cypriniformes	2
Alburnus sava	Leuciscidae	Cypriniformes	6
Alosa immaculata	Alosidae	Clupeiformes	59
Alosa tanaica	Alosidae	Clupeiformes	16
Alpinocottus poecilopus	Cottidae	Perciformes	40
Ameiurus melas	Ictaluridae	Siluriformes	334
Ameiurus nebulosus	Ictaluridae	Siluriformes	111
Anguilla anguilla	Anguillidae	Anguilliformes	395
Atherina boyeri	Atherinidae	Atheriniformes	4
Babka gymnotrachelus	Gobiidae	Gobiiformes	889
Ballerus ballerus	Leuciscidae	Cypriniformes	174
Ballerus sapa	Leuciscidae	Cypriniformes	477
Barbatula barbatula	Nemacheilidae	Cypriniformes	3193
Barbus balcanicus	Cyprinidae	Cypriniformes	1737
Barbus barbus	Cyprinidae	Cypriniformes	5419
Barbus carpathicus	Cyprinidae	Cypriniformes	14
Barbus petenyi	Cyprinidae	Cypriniformes	213
Benthophilus nudus	Gobiidae	Gobiiformes	6
Benthophilus stellatus	Gobiidae	Gobiiformes	15
Blicca bjoerkna	Leuciscidae	Cypriniformes	2199
Carassius auratus	Cyprinidae	Cypriniformes	522
Carassius carassius	Cyprinidae	Cypriniformes	186
Carassius gibelio	Cyprinidae	Cypriniformes	2434
Chelon saliens	Mugilidae	Mugiliformes	3
Chondrostoma nasus	Leuciscidae	Cypriniformes	5253
Clupeonella cultriventris	Ehiravidae	Clupeiformes	10
Cobitis elongata	Cobitidae	Cypriniformes	628
Cobitis elongatoides	Cobitidae	Cypriniformes	1711
Cobitis strumicae	Cobitidae	Cypriniformes	4
Cobitis taenia	Cobitidae	Cypriniformes	70
Cottus gobio	Cottidae	Perciformes	3639

Species	Family	Order	Occurrences
Ctenopharyngodon idella	Xenocyprididae	Cypriniformes	143
Cyprinus carpio	Cyprinidae	Cypriniformes	2349
Esox lucius	Esocidae	Esociformes	2290
Eudontomyzon danfordi	Petromyzontidae	Petromyzontiformes	57
Eudontomyzon mariae	Petromyzontidae	Petromyzontiformes	347
Gambusia holbrooki	Poeciliidae	Cyprinodontiformes	4
Gasterosteus aculeatus	Gasterosteidae	Perciformes	449
Gobio gobio	Gobionidae	Cypriniformes	1725
Gobio obtusirostris	Gobionidae	Cypriniformes	2600
Gymnocephalus baloni	Percidae	Perciformes	293
Gymnocephalus cernua	Percidae	Perciformes	964
Gymnocephalus schraetser	Percidae	Perciformes	872
Hucho hucho	Salmonidae	Salmoniformes	2085
Huso huso	Acipenseridae	Acipenseriformes	39
Hypophthalmichthys molitrix	Xenocyprididae	Cypriniformes	415
Hypophthalmichthys nobilis	Xenocyprididae	Cypriniformes	226
Ictalurus punctatus	Ictaluridae	Siluriformes	1
Knipowitschia caucasica	Gobiidae	Gobiiformes	6
Lampetra planeri	Petromyzontidae	Petromyzontiformes	45
Lepomis gibbosus	Centrarchidae	Centrarchiformes	1628
Leucaspius delineatus	Leuciscidae	Cypriniformes	98
Leuciscus aspius	Leuciscidae	Cypriniformes	2825
Leuciscus idus	Leuciscidae	Cypriniformes	1661
Leuciscus leuciscus	Leuciscidae	Cypriniformes	1216
Lota lota	Lotidae	Gadiformes	770
Micropterus salmoides	Centrarchidae	Centrarchiformes	16
Misgurnus fossilis	Cobitidae	Cypriniformes	220
Mugil cephalus	Mugilidae	Mugiliformes	4
Neogobius fluviatilis	Gobiidae	Gobiiformes	1115
Neogobius melanostomus	Gobiidae	Gobiiformes	2925
Oncorhynchus mykiss	Salmonidae	Salmoniformes	2946
Pelecus cultratus	Leuciscidae	Cypriniformes	218
Perca fluviatilis	Percidae	Perciformes	3326
Perccottus glenii	Odontobutidae	Gobiiformes	104
Petroleuciscus borysthenicus	Leuciscidae	Cypriniformes	6
Phoxinus lumaireul	Leuciscidae	Cypriniformes	3
Phoxinus phoxinus	Leuciscidae	Cypriniformes	1350
Platichthys flesus	Pleuronectidae	Pleuronectiformes	2
Ponticola eurycephalus	Gobiidae	Gobiiformes	154
Ponticola kessleri	Gobiidae	Gobiiformes	1505
Ponticola syrman	Gobiidae	Gobiiformes	1
Proterorhinus marmoratus	Gobiidae	Gobiiformes	173
Proterorhinus semilunaris	Gobiidae	Gobiiformes	434
Pseudorasbora parva	Gobionidae	Cypriniformes	1640
Pungitius platygaster	Gasterosteidae	Perciformes	11
Pungitius pungitius	Gasterosteidae	Perciformes	4
Rhodeus amarus	Acheilognathidae	Cypriniformes	3684

Species	Family	Order	Occurrences
Romanogobio kesslerii	Gobionidae	Cypriniformes	415
Romanogobio skywalkeri	Gobionidae	Cypriniformes	7
Romanogobio uranoscopus	Gobionidae	Cypriniformes	399
Romanogobio vladykovi	Gobionidae	Cypriniformes	2303
Rutilus meidingeri	Leuciscidae	Cypriniformes	11
Rutilus rutilus	Leuciscidae	Cypriniformes	5236
Rutilus virgo	Leuciscidae	Cypriniformes	1542
Sabanejewia balcanica	Cobitidae	Cypriniformes	742
Sabanejewia bulgarica	Cobitidae	Cypriniformes	60
Salmo marmoratus	Salmonidae	Salmoniformes	1
Salmo trutta	Salmonidae	Salmoniformes	6362
Salvelinus alpinus	Salmonidae	Salmoniformes	56
Salvelinus fontinalis	Salmonidae	Salmoniformes	629
Sander lucioperca	Percidae	Perciformes	1965
Sander volgensis	Percidae	Perciformes	203
Scardinius erythrophthalmus	Leuciscidae	Cypriniformes	1182
Silurus glanis	Siluridae	Siluriformes	1336
Squalius cephalus	Leuciscidae	Cypriniformes	9906
Syngnathus abaster	Syngnathidae	Syngnathiformes	120
Telestes souffia	Leuciscidae	Cypriniformes	773
Thymallus thymallus	Salmonidae	Salmoniformes	2395
Tinca tinca	Tincidae	Cypriniformes	809
Umbra krameri	Umbridae	Esociformes	66
Vimba vimba	Leuciscidae	Cypriniformes	2139
Zingel streber	Percidae	Perciformes	613
Zingel zingel	Percidae	Perciformes	613
